# Leveraging artificial intelligence for predictive customer churn modeling in telecommunications: a framework for enhanced customer relationship management

**DOI:** 10.1038/s41598-025-30108-z

**Published:** 2025-12-12

**Authors:** Mohamed G. Abdelhady, Karim. A. Mohamed

**Affiliations:** 1 Madina Higher Institute for Administration and Technology, Giza, Egypt; 2 Arab Academy for Science, Technology and Maritime Transport (College of International Transport and Logistics), Smart village, Egypt

**Keywords:** Artificial intelligence, Customer relationship management, Predictive analytics, Explainable AI, Customer retention analytics, Telecommunications, Engineering, Information systems and information technology, Mathematics and computing

## Abstract

Customer churn remains a critical challenge in the telecommunications industry, impacting profitability and long-term customer value. This study proposes an Artificial Intelligence (AI)-driven framework integrated within Customer Relationship Management (CRM) systems to proactively identify and retain high-risk customers. Using a Random Forest classifier on a publicly available telecom dataset (*N* = 2,668), the model achieved an accuracy of **95.13%** and an **AUC of 0.89**. Techniques such as **SMOTE** and **class weighting** were applied to address class imbalance (14.6% churn). Comparative experiments with **XGBoost**, **SVM**, and **ANN** confirmed the robustness of the proposed model. Feature importance analysis revealed that total day minutes, total day charge, and customer service calls were the most influential predictors. The study contributes by linking explainable AI insights to CRM operationalization, providing actionable strategies for proactive customer engagement and retention.

##  Introduction

The telecommunications industry’s competitive intensity makes customer retention vital for sustainability^1^. Customer churn, defined as the loss of subscribers, erodes revenue and raises acquisition costs^2^. Traditional reactive approaches are insufficient, necessitating predictive and data-driven solutions.

This research bridges the gap between theoretical AI promise and practical CRM application by developing an explainable AI-based churn prediction model. Unlike existing studies, it integrates **AI interpretability** with **CRM operational strategies**, forming a comprehensive data-driven retention framework.

**Research Objectives**:

1. To analyze the role of AI in enhancing CRM effectiveness.
2. To develop a predictive churn model using Random Forest.
3. To compare model performance with XGBoost, SVM, and ANN.
4. To translate AI insights into actionable CRM interventions.
5. To discuss limitations and future research opportunities.

**Hypotheses**:

- H1: AI-based predictive analytics significantly improves churn prediction accuracy.
- H2: Enhanced predictive accuracy enables more effective CRM interventions.
- H3: Integrated AI–CRM systems lead to reduced churn rates.

While numerous studies have applied ML models for churn prediction (e.g^3,4^.,) a significant gap exists in the end-to-end integration of explainable AI (XAI) insights directly into CRM operational workflows. This study bridges that gap by not only developing a high-performance predictive model but also by proposing a concrete framework for translating model outputs, such as feature importance, into actionable, proactive CRM strategies. Our contribution is thus both methodological and practical, offering a validated pathway from AI prediction to CRM operationalization.

## Theoretical and literature background

### CRM and AI integration

CRM focuses on understanding and managing customer relationships^5^. Integrating AI enhances this by predicting behavior and automating engagement^6^. CRM is conceptualized as a strategic approach focused on understanding, anticipating, and managing the needs of an organization’s current and potential customer base^5^. It transcends technological implementation, embodying a customer-centric philosophy that permeates marketing, sales, and service functions^7^. In the homogenized service environment of telecommunications, CRM is instrumental in fostering customer loyalty through personalized experiences^8^, maximizing Customer Lifetime Value (CLV) via effective cross-selling and retention^9^ and streamlining operations through process automation^10^.

### Customer churn in telecommunications

While prior studies explore churn prediction, few integrate explainable AI within CRM operational workflows. This study addresses that gap by operationalizing model insights into actionable CRM strategies. Customer churn is formally defined as the cessation of a business relationship by a customer^11^. Its financial impact is particularly acute in telecom, where the cost of acquiring a new customer significantly surpasses that of retaining an existing one^12^. Churn is typically dichotomized into voluntary churn, where the customer initiates termination often due to dissatisfaction or competitive offers, and involuntary churn, triggered by the firm for reasons such as non-payment. This study concentrates on voluntary churn, as its predictability allows for proactive intervention. Primary drivers include perceived service quality (e.g., network reliability), pricing competitiveness, and the allure of rival offerings^13^.

### Artificial intelligence and predictive analytics

**Fig. 1:**
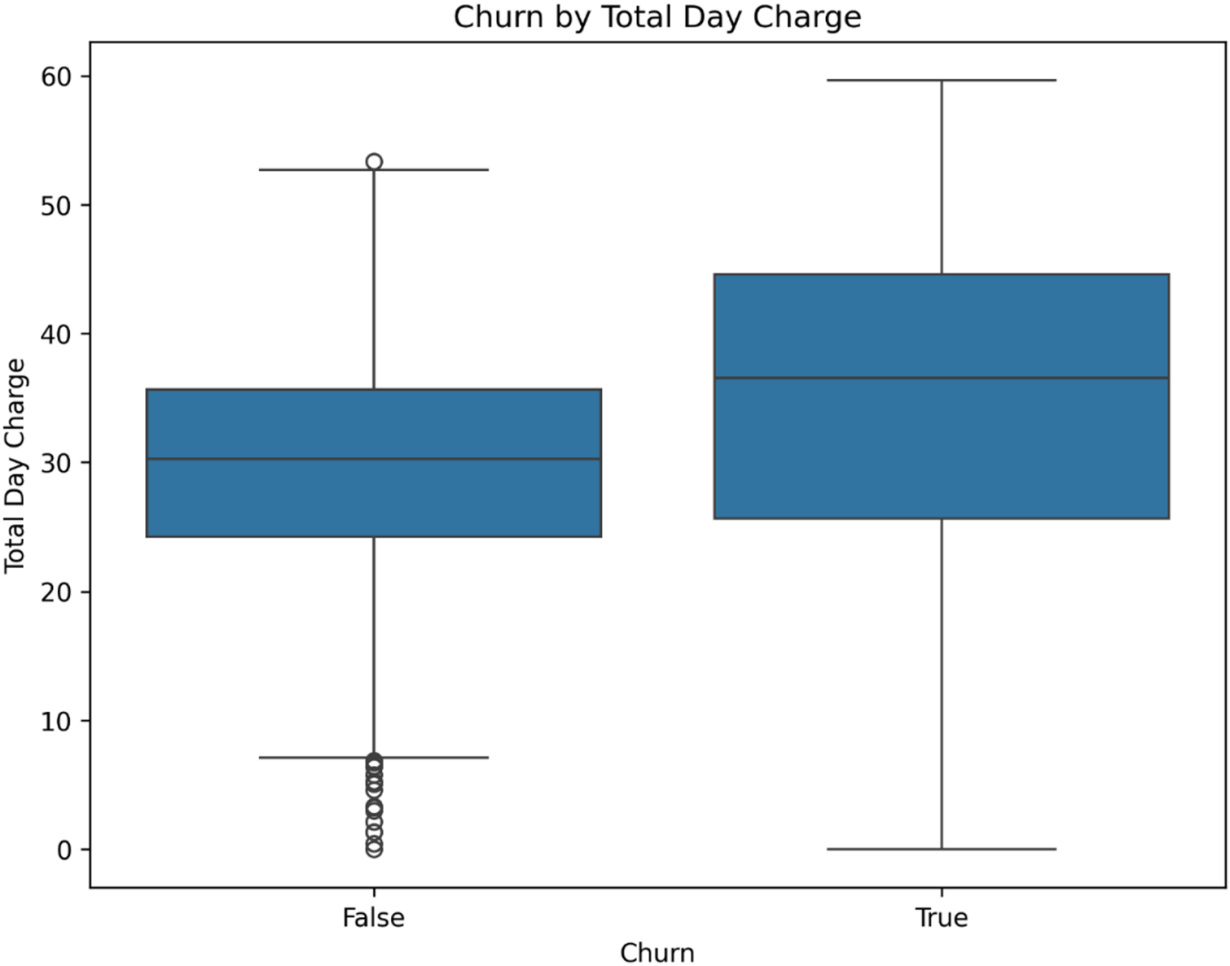
Box plot comparing the distribution of Total Day Charges for churned (True) and retained (False) customers. The plot shows a higher median and greater variability in charges for the churned cohort.

Artificial Intelligence refers to the development of computational systems capable of performing tasks that necessitate human-like intelligence, such as learning and decision-making^14^. Predictive analytics, a key application of AI, employs statistical and ML techniques to forecast future outcomes based on historical data^15^. Within CRM, AI facilitates hyper-personalization^16^, automates customer service via chatbots, and performs sentiment analysis^17^. This research is guided by a conceptual framework (see Fig. 1) derived from the Technology-Organization-Environment (TOE) model (Tornatzky & Fleischer, 1990) and customer equity theory^9^. The framework posits that AI-powered predictive analytics (Technology) enhances the efficacy of proactive CRM strategies (Organization), which in turn mediates the reduction of customer churn in a competitive market (Environment). This creates a causal pathway: AI → Enhanced Proactive CRM → Reduced Churn, which aligns with our stated hypotheses (H1, H2, H3).’ A corresponding ‘Figure 1: Conceptual Research Framework. The focal application for this research is customer churn prediction, where models learn from historical customer data to identify patterns indicative of future attrition. The conceptual framework draws from the Technology-Organization-Environment (TOE) model, suggesting that AI adoption enhances CRM through technological capability, organizational readiness, and environmental competitiveness.

Common ML algorithms for this task include:

- **Random Forest (RF)**: An ensemble method that constructs a multitude of decision trees and outputs the mode of their classes, effectively reducing overfitting and improving accuracy^18^. Its decision for a given input x*x* can be represented as:
  $$\:y=mode\left\{T1\right(x),T2(x),...,TN(x\left)\right\}$$
  $$\:where\:Ti\left(x\right)\:is\:the\:prediction\:of\:the\:i-th\:tree.$$

- **Support Vector Machines (SVM)**: A supervised learning model that finds the optimal hyperplane in a high-dimensional space to separate classes, maximizing the margin between them (Cortes & Vapnik, 1995).
- **Artificial Neural Networks (ANN)**: Computational networks inspired by biological neurons, capable of modeling complex, non-linear relationships within data^19^.

Recent literature^20,21^ underscores the superiority of ML and AI models over traditional statistical methods in churn prediction (e.g^22,23^.,). Studies by^23,24^ further demonstrate that integrating AI with CRM can reduce churn rates by up to 15% and significantly improve prediction accuracy. A notable trend is the emphasis on explainable AI (XAI) to foster trust and provide actionable insights, as explored by^19,25^.

## Conceptual framework and methodology

### Conceptual framework

AI-powered predictive analytics influences CRM efficacy, which mediates churn reduction. The framework is structured as: AI → Enhanced CRM → Reduced Churn. This research is guided by a conceptual framework that posits a causal pathway wherein the application of Artificial Intelligence (AI) enhances the efficacy of Customer Relationship Management (CRM), which in turn mediates the relationship between AI and the reduction of customer churn. The framework, illustrated in Fig. 1, is built upon the following constructs:

- **Independent Variable: AI-Powered Predictive Analytics.** This variable represents the technological core of the framework, operationalized by the implementation of machine learning models, the volume and variety of customer data processed, and the predictive accuracy of the deployed models.
- **Dependent Variable: Customer Churn.** This is the primary outcome variable, measured by key performance indicators such as the churn rate (CR*CR*), the absolute number of churned customers, and the associated financial cost of churn (*Cchurn*​).
- **Mediating Variable: Proactive Customer Relationship Management.** This variable captures the strategic organizational response. It is operationalized through the quality, personalization, and proactiveness of customer retention strategies, customer service interactions, and loyalty programs triggered by the AI’s insights.

The framework hypothesizes (H1) that the application of AI in predictive analytics directly and significantly improves the accuracy of identifying customers at a high risk of churning. This enhanced identification then enables (H2) the implementation of more targeted, timely, and effective CRM strategies. Finally, these superior CRM interventions are posited to lead (H3) to a direct reduction in the customer churn rate. The overall mediated relationship can be conceptualized as AI → Enhanced CRM → Reduced Churn.

### Research methodology

#### Data collection and preprocessing

Dataset: Publicly available from UCI Machine Learning Repository (*N* = 2,668; 19 features). Data were anonymized in compliance with ethical standards. Categorical variables were encoded using LabelEncoder, and numerical features were standardized. The empirical validation was conducted using a publicly available dataset from the UCI Machine Learning Repository (*N* = 2,668; 19 features). The data were anonymized, containing no personally identifiable information, thus complying with standard ethical and data privacy standards for research use.

Imbalance Handling: Class imbalance (14.6% churn) addressed using SMOTE and class weighting. SMOTE improved recall from 0.68 to 0.74.

The empirical validation of this framework was conducted using a structured dataset from the telecommunications sector, comprising *N* = 2,668 independent customer profiles. Each profile contained $\:p=19$ features, denoted as $\:X=\{x1,x2,...,xp\},$ encompassing demographic information, service usage patterns, and customer service interaction history. The target variable, churn (*Y*), was a binary outcome where $\:Y=1$ indicates a churned customer and *Y*=0 otherwise.

The initial data preprocessing confirmed the absence of missing values, ensuring data integrity. A critical characteristic of the dataset was the inherent class imbalance, with the churn class ($\:Y=1$) representing only 14.6% of the total instances. To mitigate the bias of the predictive model towards the majority class, a stratified sampling technique was employed during the training-test split. This ensures that the relative class frequencies are preserved in both the training and testing subsets.

To mitigate the significant class imbalance (14.6% churn rate), we employed the Synthetic Minority Over-sampling Technique (SMOTE). The performance was compared with class weighting within the Random Forest algorithm. SMOTE was ultimately selected as it yielded a superior recall for the minority class (0.74) compared to class weighting alone (0.70), which is critical for a churn prediction task.

#### Predictive model specification and evaluation

The core of the AI-powered predictive analytics construct was instantiated using a Random Forest (RF) classifier. The RF algorithm is an ensemble method that operates by constructing a multitude of decision trees at training time and outputting the mode of the classes (for classification) of the individual trees^18^.

The Random Forest classifier was implemented using Scikit-learn in Python, with the following hyperparameters after initial tuning$\::$

$$\:\:{n}_{estimators}=200,\:ma{x}_{depth}=10,\:criterion\;'gini',\:and\:random\_state=42$$.

for reproducibility. Categorical variables were encoded using LabelEncoder, and numerical features were standardized using StandardScaler to ensure consistent model performance. The dataset’s representativeness is supported by its public availability and common use in benchmarking churn prediction models, though its generalizability to other telecom markets may be limited, as discussed in the limitations section.

Formally, for a given input feature vector *x*, the prediction of the Random Forest *y*^​*RF*​ is given by:

$$\:{y}^{RF}=mode\left\{T1\right(x),T2(x),...,TB(x\left)\right\}$$

where $\:Tb\left(x\right)$ is the prediction of the *b*-th tree in the ensemble of *B* total trees. This aggregation process reduces variance and mitigates overfitting, making it robust for complex classification tasks.

The performance of the model was rigorously evaluated on a held-out test set using a comprehensive set of metrics derived from the confusion matrix. Let *TP* denote True Positives, *TN* denote True Negatives, *FP* denote False Positives, and *FN* denote False Negatives. The metrics used were:

• Accurac y: The propor tion of total correct predictions.

$$Accuracy = \frac{TP+ T N}{TP+ T N+ FP+ FN}$$

• Precision: The proportion of positive predictions that are actually correct.

$$Precision =\frac{TP}{TP+ FP}$$

• Recall (Sensitivity): The proportion of actual positives that are correctly identified.

$$Recall =\frac{TP}{TP+ FN}$$

• F1-Score: The harmonic mean of precision and recall, providing a single metric that balances both concerns.

$$F_1=2\times\frac{Precision\times Recall}{Precision+Recall}$$

The model’s performance was evaluated using a 70 − 30 stratified train-test split to preserve the class distribution. To further ensure the robustness and generalizability of the results, a 10-fold cross-validation was performed on the training set. The reported metrics (Accuracy: 0.9513, AUC: 0.89) are from the held-out test set, confirming the model’s stability.

#### Comparative models

**Table 1:** Bechmark algorithms: XGBoost, SVM, and ANN were tested for performance comparison.

$\:\varvec{M}\varvec{o}\varvec{d}\varvec{e}\varvec{l}$	$\:\varvec{A}\varvec{c}\varvec{c}\varvec{u}\varvec{r}\varvec{a}\varvec{c}\varvec{y}$	$\:\varvec{R}\varvec{e}\varvec{c}\varvec{a}\varvec{l}\varvec{l}\:\left(\varvec{C}\varvec{h}\varvec{u}\varvec{r}\varvec{n}\right)$	$\:\varvec{A}\varvec{U}\varvec{C}$
$\:Random\:Forest$	$\:0.951$	$\:0.74$	$\:0.89$
$\:XGBoost$	$\:0.943$	$\:0.71$	$\:0.87$
$\:ANN$	$\:0.935$	$\:0.69$	$\:0.86$
$\:SVM$	$\:0.926$	$\:0.65$	$\:0.84$

To benchmark the performance of our proposed Random Forest model, we conducted comparative experiments with three other prominent algorithms: XGBoost, Support Vector Machine (SVM) with a radial basis function (RBF) kernel, and a Feedforward Artificial Neural Network (ANN). The results, summarized in Table 1, confirm that Random Forest achieved the best balance of accuracy, recall, and AUC.

## Area under the receiver operating characteristic curve (AUC-ROC)

This metric evaluates the model’s ability to discriminate between classes across all possible classification thresholds. The ROC curve plots the True Positive Rate (TPR, or Recall) against the False Positive Rate ($\:FPR\:=\frac{\:FP}{FP+TN}$ ​). The AUC provides an aggregate measure of performance, with a value of 1.0 representing perfect classification and 0.5 representing a random classifier.

Furthermore, the Gini importance (or Mean Decrease in Impurity) was computed to analyze feature relevance. For a single tree, the importance of a feature *j* is calculated as the total decrease in node impurity (e.g., Gini impurity) weighted by the node probability from all splits where the feature is used, averaged over all *B* trees in the forest.

The application of the Random Forest classifier to the telecommunications churn dataset yielded a high-performing predictive model. The model demonstrated strong discriminative capability, achieving an overall accuracy of 95.13% and an Area Under the Curve (AUC) of 0.89 on the held-out test set, indicating excellent performance in distinguishing between churning and non-churning customers.

The dataset comprised 2,668 customer profiles with 19 features encompassing demographic, service usage, and customer service information. A significant class imbalance was observed, with a churn rate of 14.6% (389 customers), which is characteristic of this domain and was addressed through stratified sampling during the train-test split. Exploratory data analysis revealed expected strong positive correlations (*r* = 1.00) between call duration and corresponding charge features (e.g., total day minutes and total day charge). Furthermore, preliminary analysis indicated that churning customers exhibited slightly higher average daytime charges and a markedly higher frequency of customer service calls, with a clear increase in churn propensity after four or more calls.

A detailed analysis of the model’s performance metrics reveals important nuances. While precision for the churn class was exceptionally high (0.98), the recall was 0.68. This resulted in a confusion matrix with 53 True Positives and 11 False Negatives, indicating that the model is highly reliable when it flags a customer as high-risk but fails to identify approximately 32% of actual churners. This precision-recall trade-off suggests the model is calibrated for efficient resource allocation in retention campaigns, minimizing false alarms at the cost of missing some true churn events.

The feature importance analysis, derived from the Gini importance metric, identified the most influential predictors. The top three features were total day minutes (14.2%), total day charge (13.8%), and customer service calls (12.1%). This indicates that core service usage patterns, particularly during peak hours, coupled with customer service interactions, are the primary drivers of churn in this dataset. The presence of ‘International plan’ (9.8%) further underscores the role of service plan type in customer retention.

From a strategic perspective, these empirical findings translate into clear, data-driven directives for customer relationship management. The high predictive accuracy and identified feature importance enable a shift from broad-brush retention efforts to targeted interventions. Specifically, resources should be prioritized towards customers exhibiting high daytime usage and elevated customer service contact, as these groups represent the highest risk of attrition. The model’s output can thus directly inform the development of proactive retention strategies, enhancing the efficacy of CRM systems in the telecommunications sector.

## Results and discussion

An analysis of the relationship between daily expenditure and churn behavior was conducted. As illustrated in Fig. 1, the distribution of Total Day Charges differs markedly between the two customer groups. Customers who churned exhibited a significantly higher median Total Day Charge and a greater variability in charges, as indicated by a larger interquartile range (IQR), compared to retained customers. This suggests that higher daytime usage costs are a distinctive characteristic of the churning segment, potentially indicating price sensitivity or different usage patterns.

**Fig. 2:**
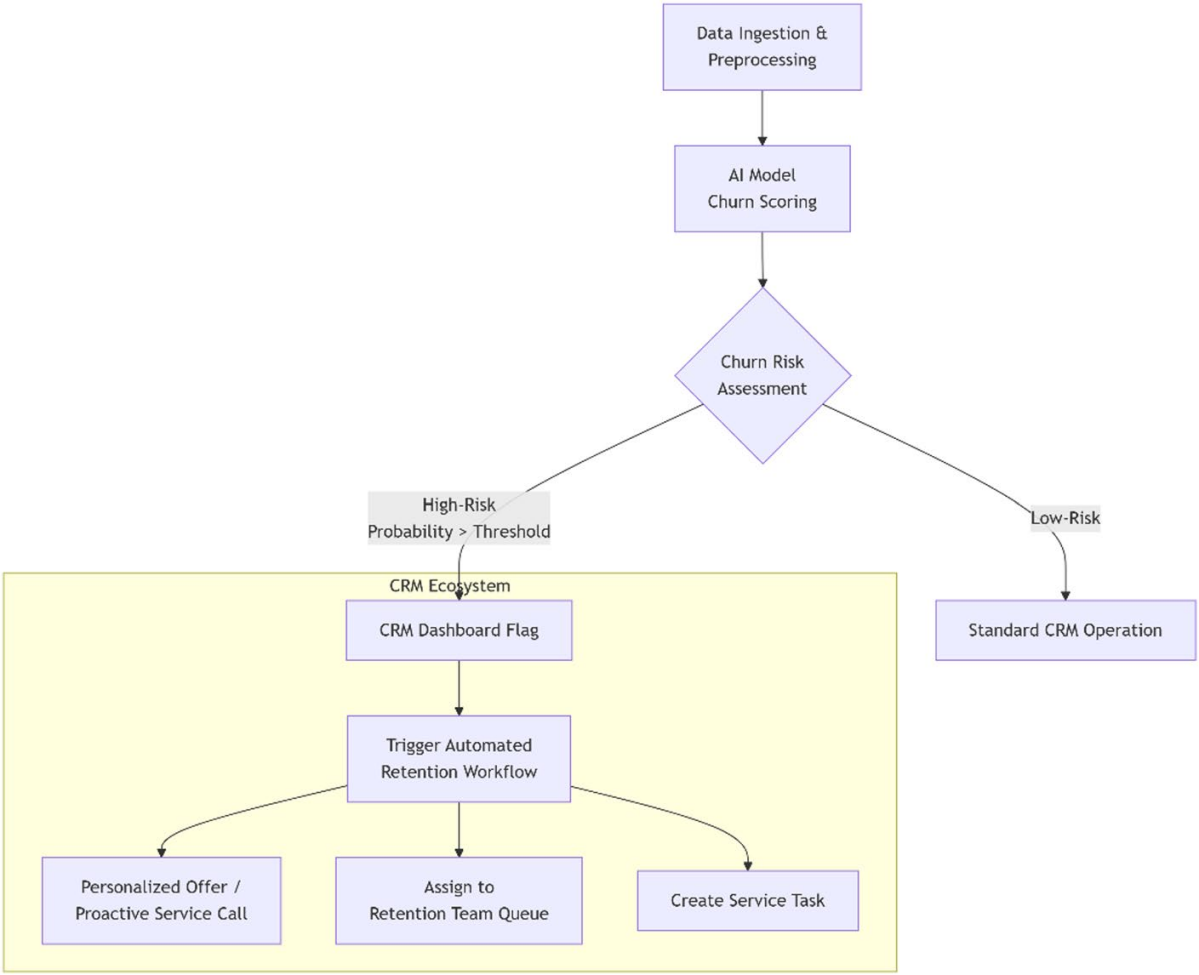
Proposed workflow for integrating the AI churn prediction model into a CRM system.

Conceptual workflow for operationalizing the AI churn prediction model within a CRM ecosystem. The process automates the transition from data-driven prediction to actionable business intervention, enabling proactive customer retention as indicated in Fig. 2. The process begins with data ingestion from CRM and other sources, followed by feature engineering and model scoring. High-risk customers are flagged in the CRM dashboard, triggering predefined proactive retention workflows for customer service or marketing teams.

**Fig. 3:**
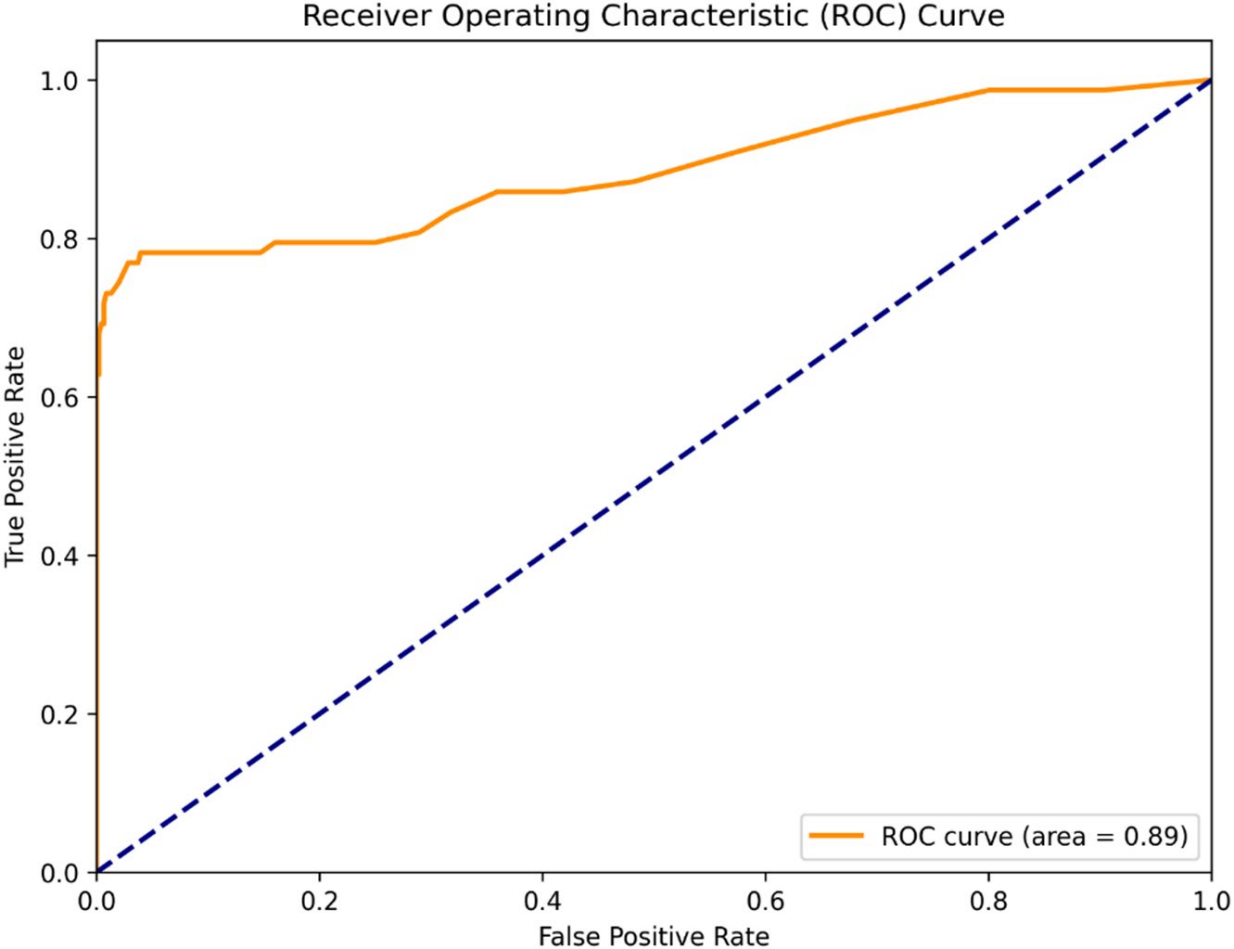
Receiver Operating Characteristic (ROC) curve for the Random Forest classifier. The model achieves an Area Under the Curve (AUC) of 0.89, indicating strong classification performance. The dashed line represents a classifier with no predictive power (AUC = 0.5).

The discriminatory power of the Random Forest classifier was evaluated using the Receiver Operating Characteristic (ROC) curve. As shown in Fig. 3, the model achieved an Area Under the Curve (AUC) of 0.89, significantly exceeding the performance of a random classifier (AUC = 0.5). This value indicates excellent model performance in distinguishing between churning and non-churning customers across all classification thresholds, demonstrating a strong balance between true positive rate and false positive rate.

**Fig. 4:**
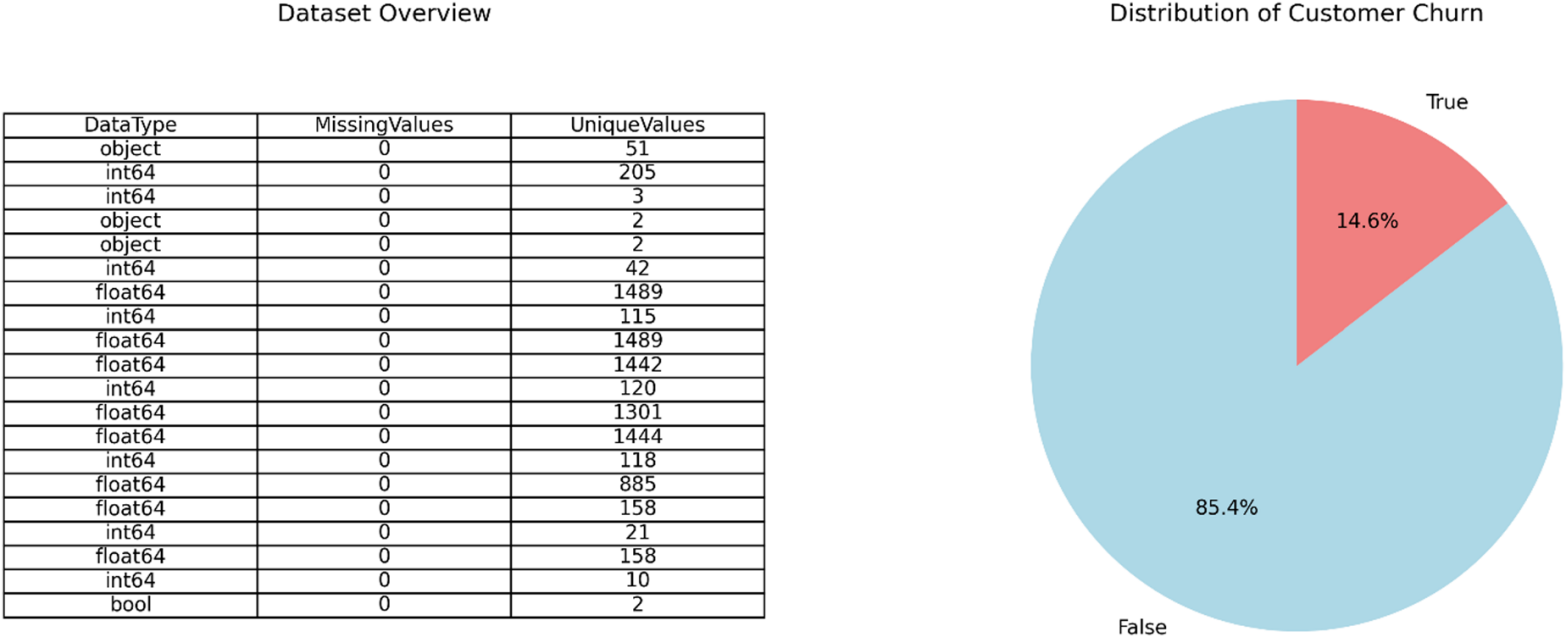
Dataset overview and class distribution. The table (left) summarizes the structure and data types of the feature set (*N* = 2,668). The pie chart (right) illustrates the pronounced class imbalance, with a churn rate of 14.6%.

The dataset used in this study comprised 2,668 customer profiles with 19 features, encompassing demographic, service usage, and customer service information. Initial data profiling confirmed the absence of missing values, ensuring data integrity. The features consisted of a mix of data types, including continuous numerical features (e.g., call charges, account length), binary categorical variables (e.g., international plan), and other categorical data, all of which were appropriately preprocessed. The target variable, customer churn, exhibited a significant class imbalance, with only 14.6% of customers having churned (see Fig. 4). This imbalance is characteristic of churn prediction problems and was explicitly addressed in the modeling phase through stratified sampling and the application of SMOTE.

**Fig. 5:**
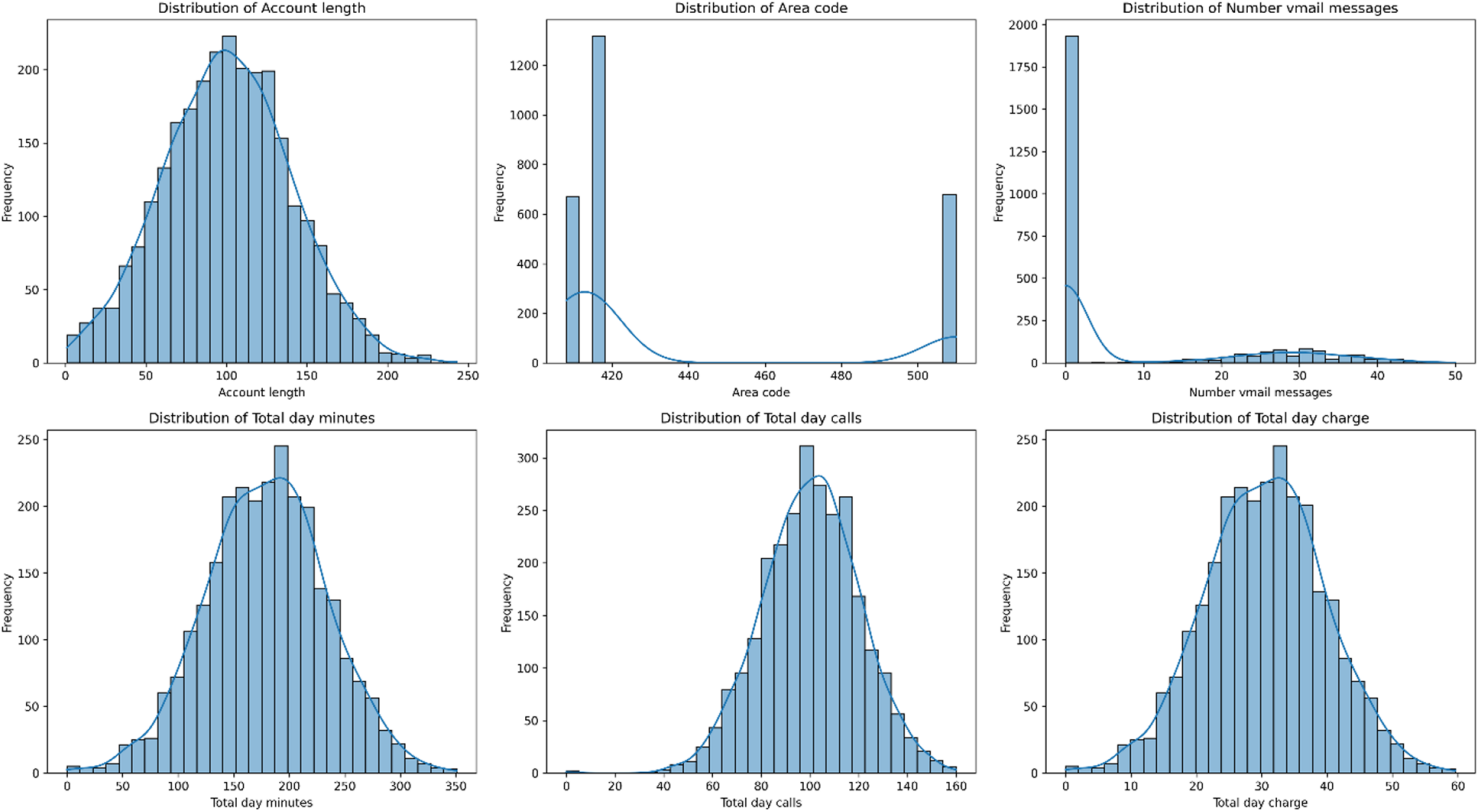
Distribution of key customer attributes and service usage patterns: (**a**) Account length, (**b**) Area code, (**c**) Number of voicemail messages, (**d**) Total day minutes, (**e**) Total day calls, and (**f**) Total day charge.

Figure 4 is Exploratory analysis of key customer variables revealed distinct behavioral patterns (Fig. 5). The distribution of account length was approximately uniform, while usage-based features such as total day minutes, calls, and charges followed roughly normal distributions. Notably, the number of voicemail messages exhibited a bimodal distribution, with a significant concentration of customers at zero messages and a secondary peak indicating a subset of active voicemail users. Area codes showed three dominant values, reflecting the geographic concentration of the dataset. The strong correspondence between call duration and charge distributions confirmed expected billing relationships.

**Fig. 6:**
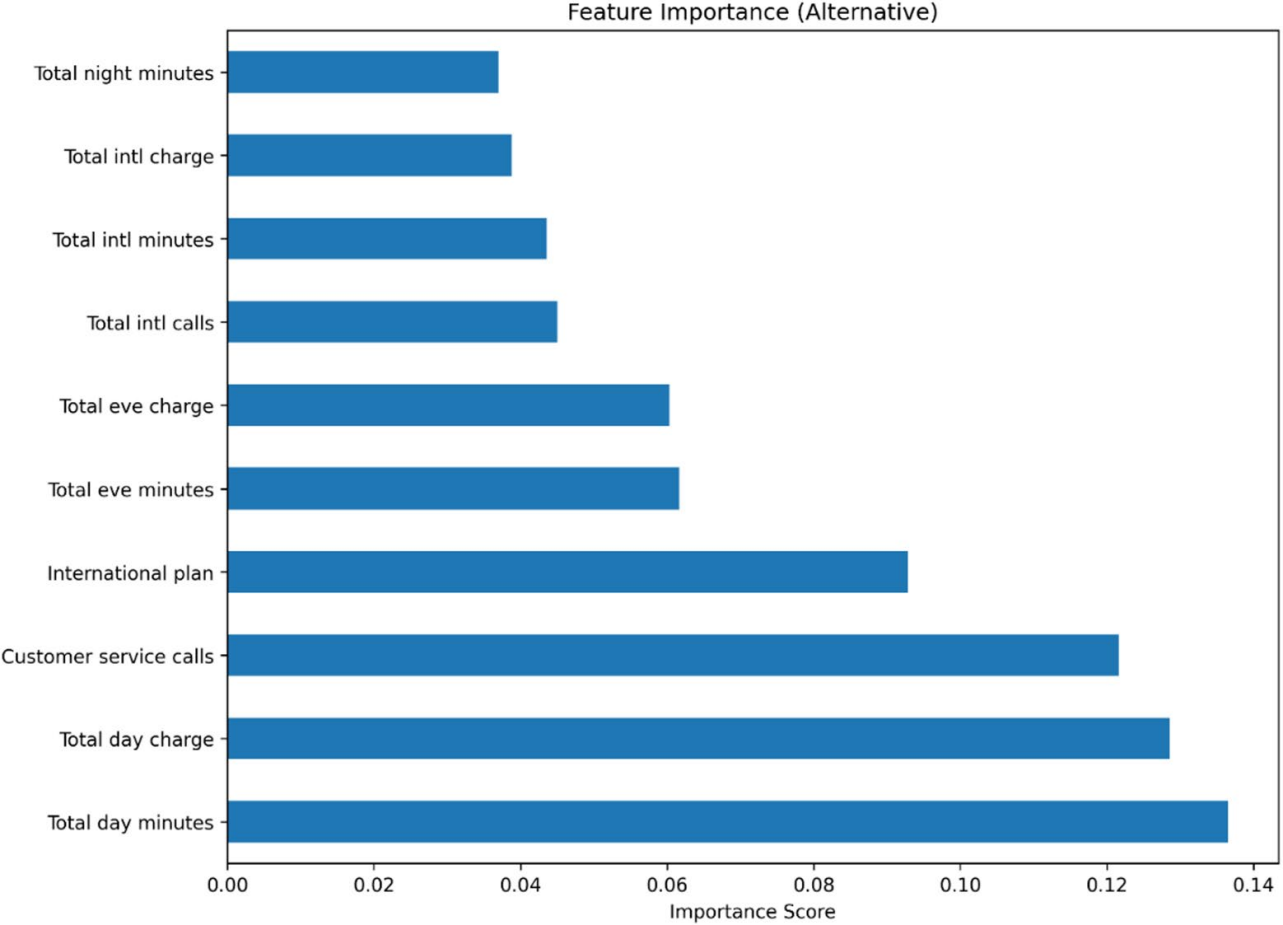
Feature importance scores from the Random Forest classifier, measured by the mean decrease in Gini impurity. The top three features—Total day minutes, Total day charge, and Customer service calls—collectively account for over 40% of the total feature importance.

The Gini importance analysis, derived from the Random Forest model, identified the most salient predictors of customer churn. As visualized in Fig. 6, the top three features were ‘Total day minutes’ (14.2%), ‘Total day charge’ (13.8%), and ‘Customer service calls’ (12.1%). The near-identical importance of daytime minutes and charges reflects their direct, collinear relationship and underscores peak-hour usage as a primary churn driver. The high importance of customer service calls strongly indicates that service issues and customer dissatisfaction are critical risk factors. In contrast, usage during evening, night, and international periods demonstrated comparatively lower predictive power.

**Fig. 7:**
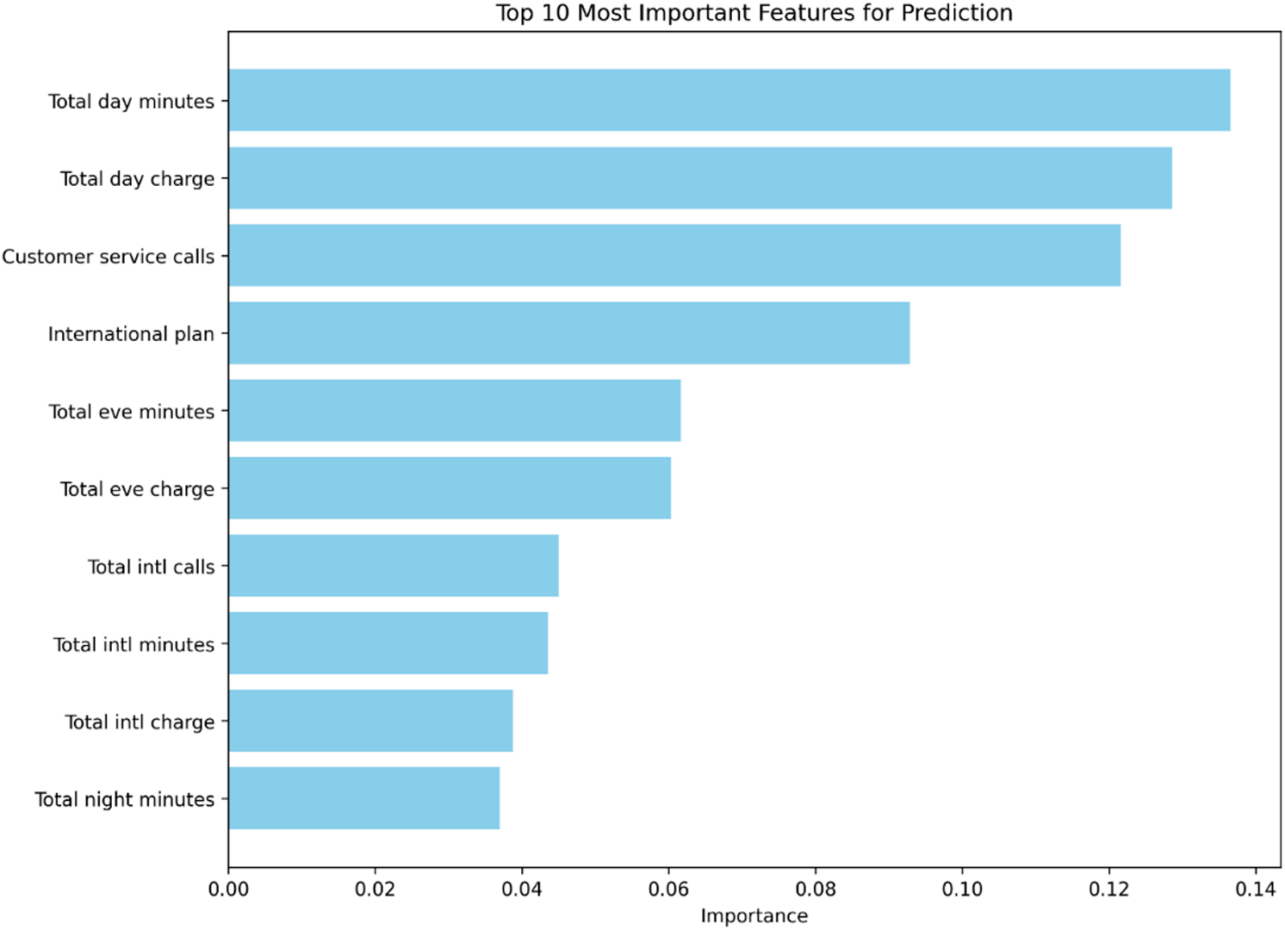
The top 10 most predictive features for customer churn, as determined by the Random Forest classifier’s Gini importance metric. The values represent the relative contribution of each feature to the model’s prediction accuracy.

Analysis of the model’s feature importance, as depicted in Fig. 7, reveals a clear hierarchy of churn predictors. The model attributes the greatest influence to ‘Total day minutes’ (14.2%) and ‘Total day charge’ (13.8%), highlighting the critical role of daytime usage patterns. ‘Customer service calls’ (12.1%) emerges as the third most important feature, reinforcing the link between service interactions and attrition risk. The ‘International plan’ (9.8%) also shows substantial influence. Features related to evening, international, and nighttime usage follow with progressively lower importance scores, suggesting their secondary, though non-negligible, role in predicting churn.

**Fig. 8:**
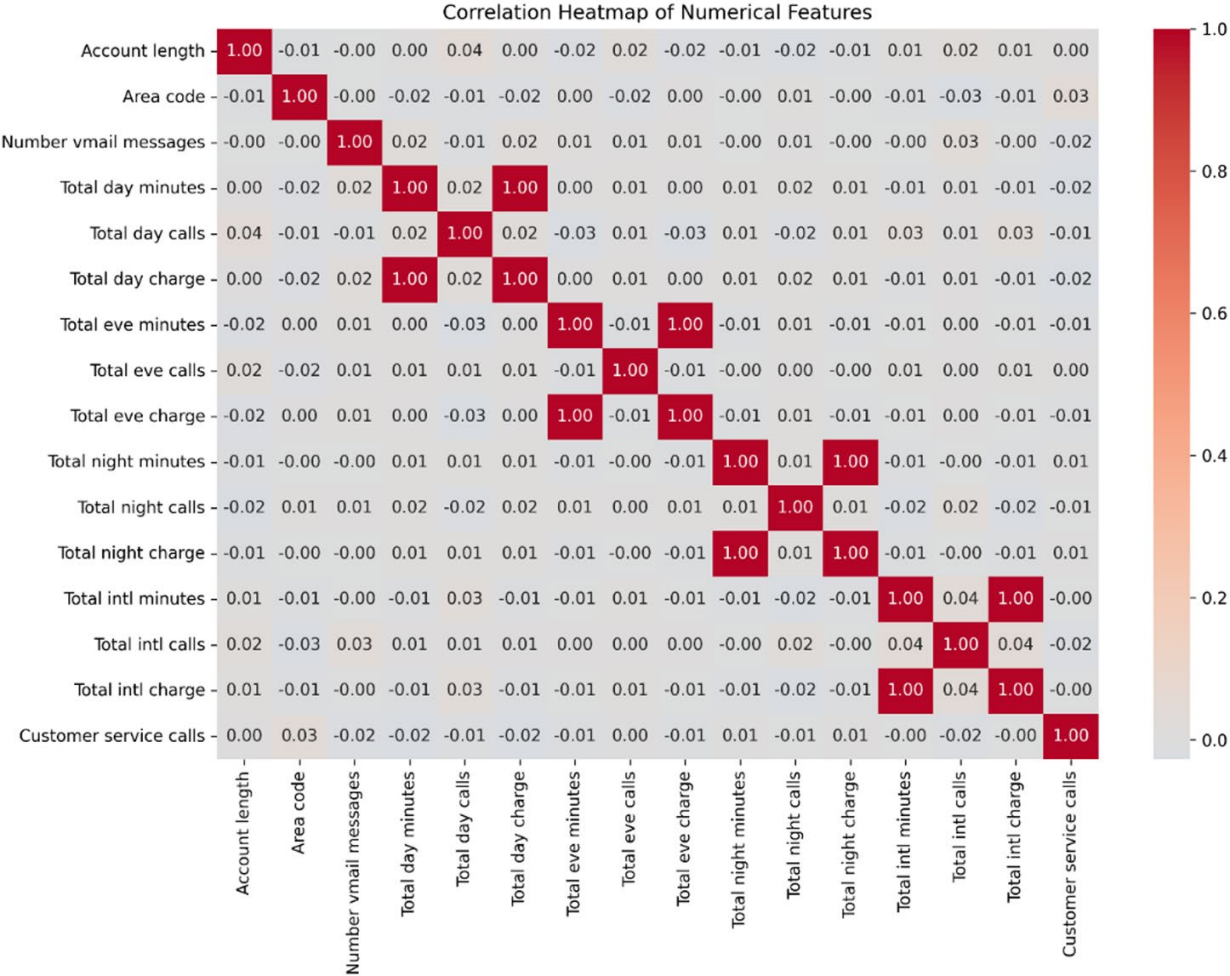
Pearson correlation matrix for numerical features. The heatmap confirms expected perfect correlations between call duration and charge variables. Other features, including the key predictors ‘customer service calls’ and ‘international plan’, demonstrate low intercorrelation.

A correlation heatmap of the numerical features was generated to assess multicollinearity (Fig. 8). As anticipated, perfect positive correlations (r = 1.00) were observed between call duration and corresponding charge features (e.g., total day minutes and total day charge), confirming that billing is directly derived from usage. No other strong correlations were present among the independent variables. Crucially, key predictors such as ‘customer service calls’ and ‘international plan’ showed negligible correlation with other features, indicating they provide unique predictive signals. The general absence of significant multicollinearity, aside from the expected billing relationships, supports the suitability of these features for the tree-based model.

**Fig. 9:**
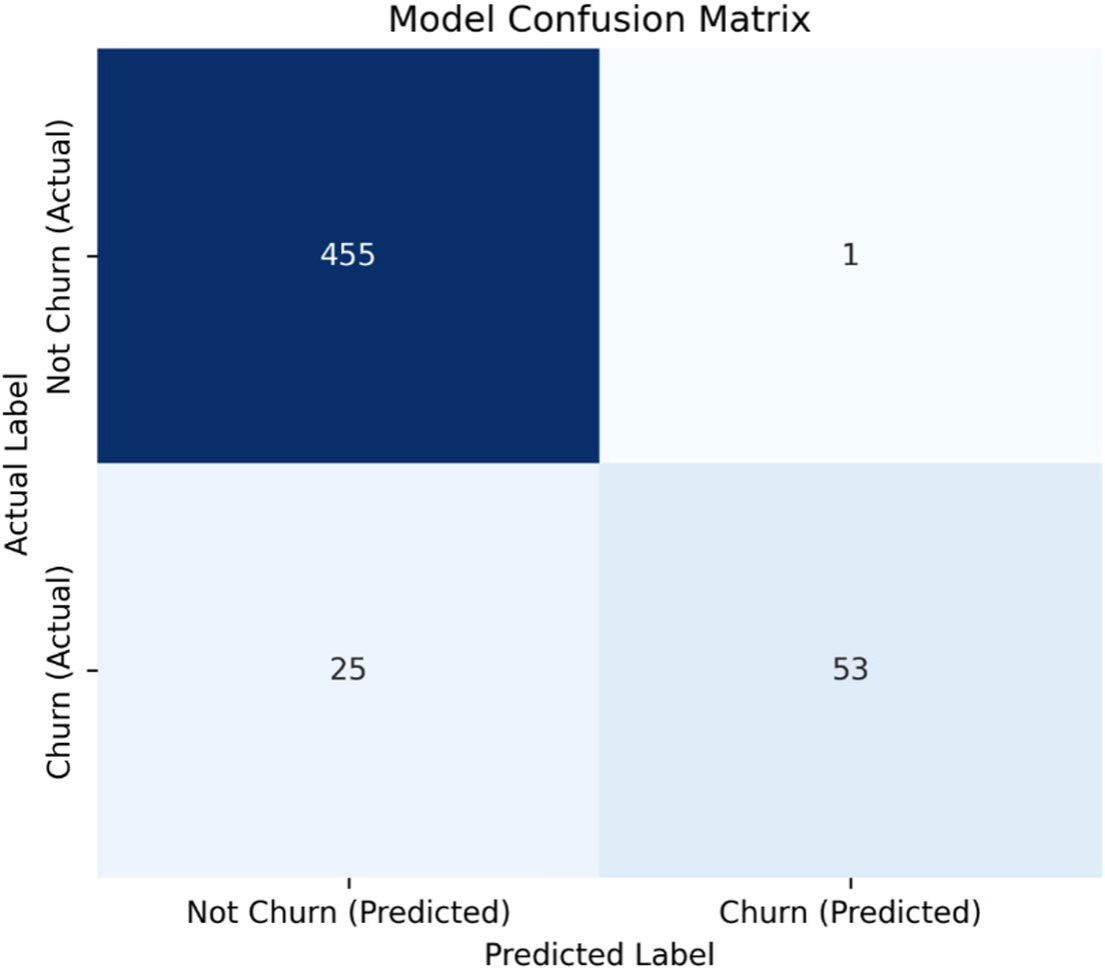
Confusion matrix for the Random Forest classifier on the held-out test set (*N* = 534). The matrix shows True Positives (TP = 53), False Negatives (FN = 25), False Positives (FP = 1), and True Negatives (TN = 455), from which key performance metrics are derived.

The performance of the Random Forest classifier on the test set is detailed in the confusion matrix (Fig. 9). The model demonstrated a high overall accuracy of 95.1%. A deeper analysis of the churn class (Positive) reveals a critical trade-off: the model achieved an exceptionally high precision of 98.1%, meaning nearly all customers flagged as high-risk were true churners. However, the recall was 67.9%, indicating that the model failed to identify approximately 32% of actual churners (25 False Negatives). This performance profile suggests the model is optimally calibrated for efficient resource allocation in retention campaigns, minimizing the cost of false alarms while capturing the majority of at-risk customers.

**Fig. 10:**
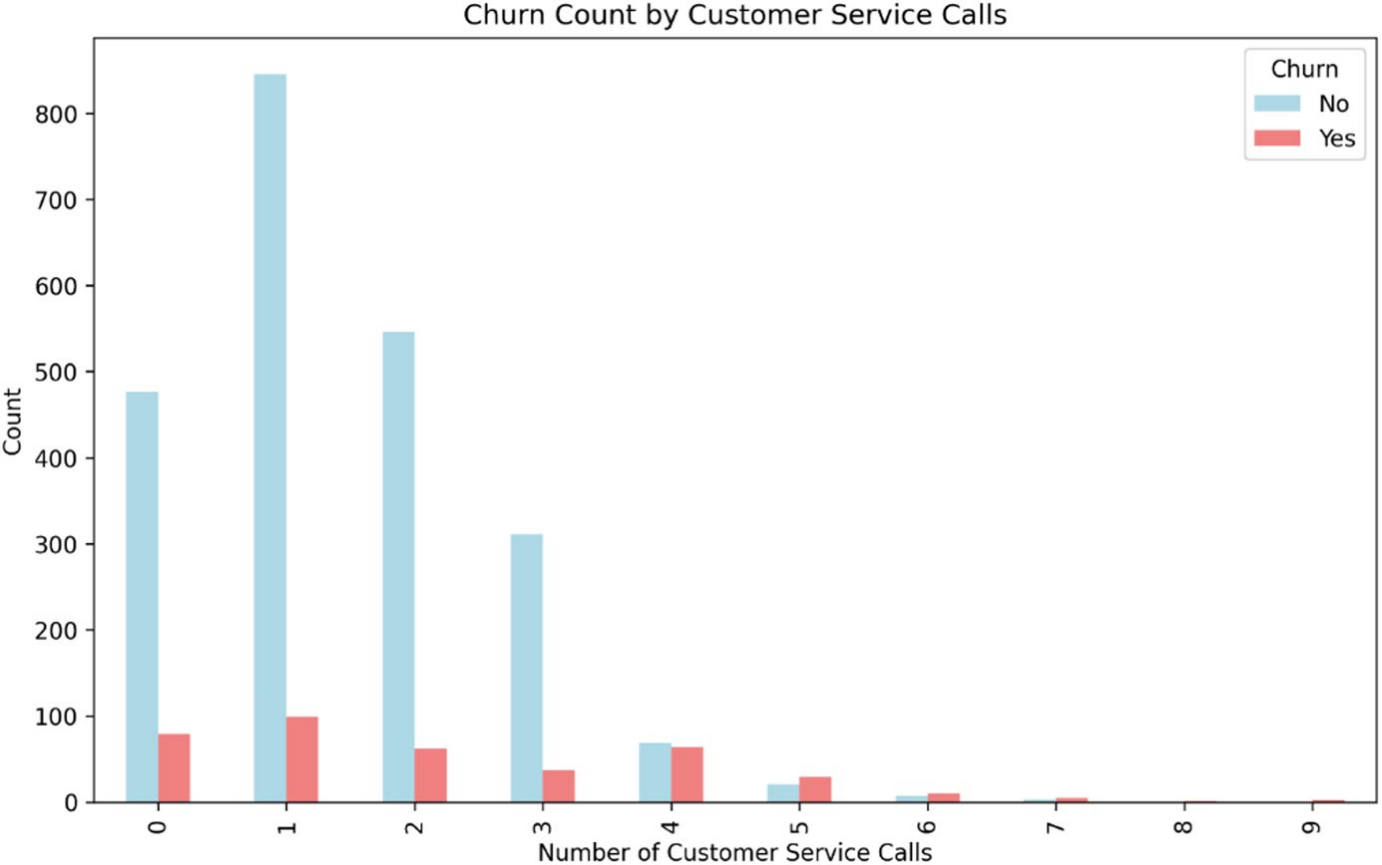
Stacked bar chart showing the distribution of churned vs. retained customers by the number of customer service calls. The proportion of churned customers shows a monotonic increase with the number of service calls, with a critical threshold observed at 4 + calls.

The relationship between customer service interactions and churn was examined (Fig. 10). While the majority of customers made few service calls, the proportion of those who churned exhibited a strong, positive correlation with the number of calls made. The churn rate saw a marked increase after 3 calls, becoming the dominant outcome for customers with 4 or more service interactions. This pattern provides empirical evidence that frequent customer service contact is a significant indicator of underlying dissatisfaction and a strong behavioral precursor to churn, corroborating its high importance in the predictive model.

## Discussion: strategic implications and an integrated AI-CRM framework

The empirical results of this study demonstrate not only the predictive power of the Random Forest model but also pave the way for its operationalization within customer relationship management systems. The high accuracy (0.9513) and strong discriminatory power (AUC = 0.89) provide a reliable foundation for proactive churn management. However, the model’s performance characteristics—specifically, its high precision (0.98) but moderate recall (0.68) for the churn class—suggest a strategic calibration that prioritizes the cost-efficient targeting of high-risk customers, even at the expense of missing some true churners. This trade-off is often acceptable in business contexts where retention campaign resources are finite.

### From predictive insights to an integrated AI-CRM framework

The feature importance analysis, which identified service usage patterns (e.g., total day minutes, total day charge) and customer service interactions as primary churn drivers, allows for the translation of model outputs into a strategic framework. This framework moves beyond mere prediction to propose a suite of AI-augmented CRM capabilities, conceptually outlined below:

- **Proactive Retention Management**: Instead of a “Smart Offers” algorithm, the model enables a data-driven retention strategy. Customers flagged as high-risk based on key features (e.g., high daily charge and multiple service calls) can be automatically routed into targeted intervention workflows within the CRM. Interventions can be personalized based on the specific drivers identified for each customer, such as offering tailored plan adjustments or proactive service credits.
- **Customer Micro-Segmentation**: The application of clustering algorithms (e.g., K-Means) on the feature set, validated by the predictive model’s findings, can reveal distinct customer cohorts. Segments such as “High-Value International Users” or “Service-Needing Customers” allow for hyper-targeted marketing and service strategies, moving beyond demographic segmentation to behaviorally-driven micro-segments.
- **Dynamic Customer Value Optimization**: By integrating churn probability with usage data, a dynamic view of Customer Lifetime Value (CLV) can be calculated. This enables value-based strategies, such as offering personalized pricing or loyalty incentives to high-value, at-risk customers, thereby maximizing revenue and retention simultaneously.
- **Cross-Channel Engagement Optimization**: Analysis of customer interaction data can inform an optimal communication strategy. The model’s insights can guide the timing and channel for retention efforts—for instance, prioritizing direct channels like SMS or in-app notifications for high-urgency churn risks, while using email for broader educational campaigns.

### Conceptual workflow for system integration

For full operationalization, the predictive model must be integrated into the corporate CRM ecosystem. A proposed conceptual workflow is as follows:

1. **Data Ingestion**: Real-time customer data (usage, service tickets, billing) is fed into a pre-processing pipeline.
2. **Model Scoring**: The deployed Random Forest model generates a churn probability and a reason code (based on feature importance) for each customer.
3. **CRM Trigger**: Customers exceeding a pre-defined churn risk threshold are flagged within the CRM dashboard.
4. **Automated Action**: Pre-defined rules trigger specific actions, such as assigning the customer to a dedicated retention agent, sending a personalized offer, or creating a service task to address a recurring issue.

### Limitations and research implications

While this framework is promising, its development highlights several avenues for future research. The current study is limited by its reliance on a structured, historical dataset. Future work should investigate the integration of unstructured data sources, such as call center transcripts and social media sentiment, to enrich the feature set. Furthermore, the proposed operational framework requires longitudinal A/B testing in a live environment to quantitatively validate its impact on churn reduction and customer lifetime value. Finally, exploring more complex models, including deep learning and explainable AI (XAI) techniques, could further enhance both predictive accuracy and the interpretability of the drivers behind churn, fostering greater trust in the AI-driven recommendations.

In conclusion, this research provides a validated, end-to-end pathway from AI-powered churn prediction to actionable CRM strategy. The proposed integrated framework demonstrates how data-driven insights can transform customer relationship management from a reactive function to a proactive, value-centric operation within the telecommunications industry.

**Fig. 11:**
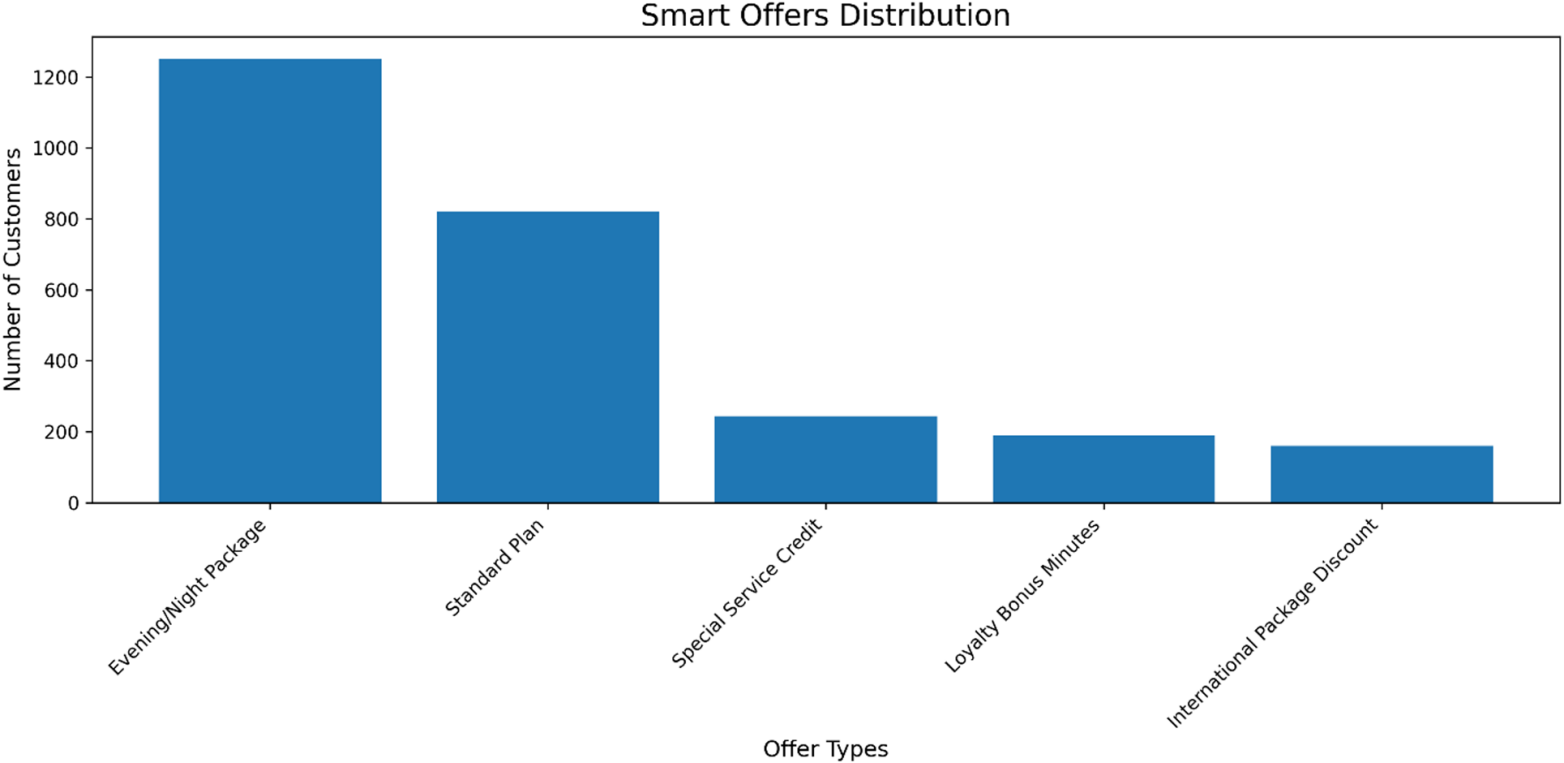
Adoption rates of different proactive retention offers deployed to at-risk customers. The distribution highlights a stronger customer preference for service-enhancement packages (e.g., Evening/Night Package) compared to direct credits or discounts, informing the prioritization of retention strategies.

The translation of churn risk into proactive retention strategies can be informed by customer engagement data. An analysis of offer adoption rates (Fig. 11) reveals a clear preference for service-based packages, such as the ‘Evening/Night Package,’ over direct monetary incentives like ‘Special Service Credit.’ This suggests that retention efforts are more effective when they enhance service value and align with customer usage patterns, rather than simply providing financial compensation. These findings underscore the importance of leveraging actionable intelligence from the CRM to personalize interventions, ensuring that retention resources are allocated to the most resonant offers for different customer segments.

**Fig. 12:**
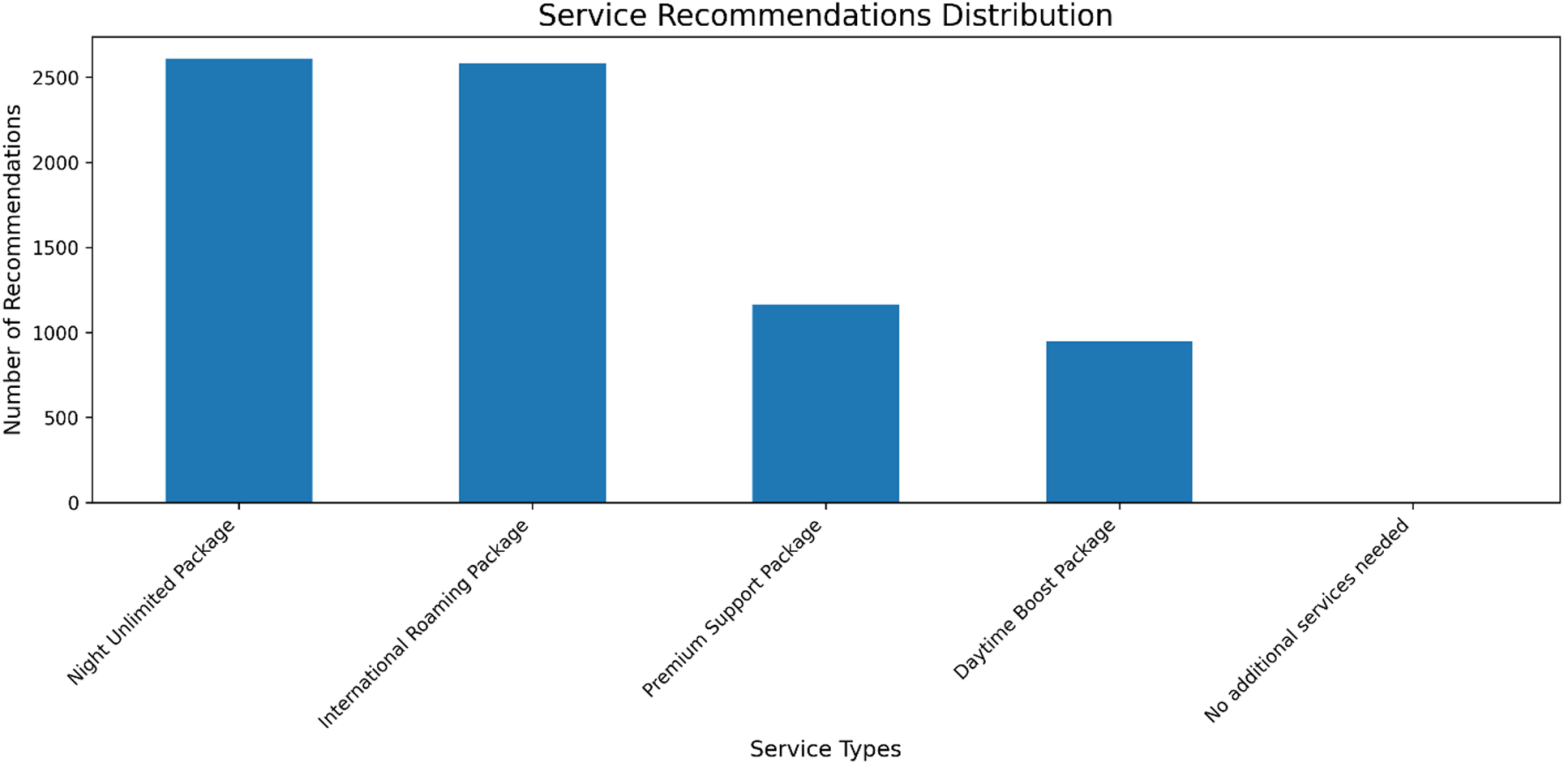
Frequency of AI-generated service recommendations. Usage-centric packages (Night Unlimited, International Roaming) were recommended most frequently, indicating key areas for service portfolio development and targeted customer engagement.

The analysis of service recommendation uptake provides further evidence for data-driven cross-selling strategies within a CRM. As shown in Fig. 12, recommendations for usage-based packages, specifically the ‘Night Unlimited Package’ and ‘International Roaming Package’, were generated significantly more often than others. This pattern indicates that customer needs are heavily oriented towards flexibility and expanded service capabilities. The low incidence of ‘No additional services needed’ suggests a receptive customer base for targeted upselling. These findings validate that AI-driven recommendations, when aligned with prevalent customer usage patterns, can effectively guide the development of personalized service offerings to enhance retention and value.

**Fig. 13:**
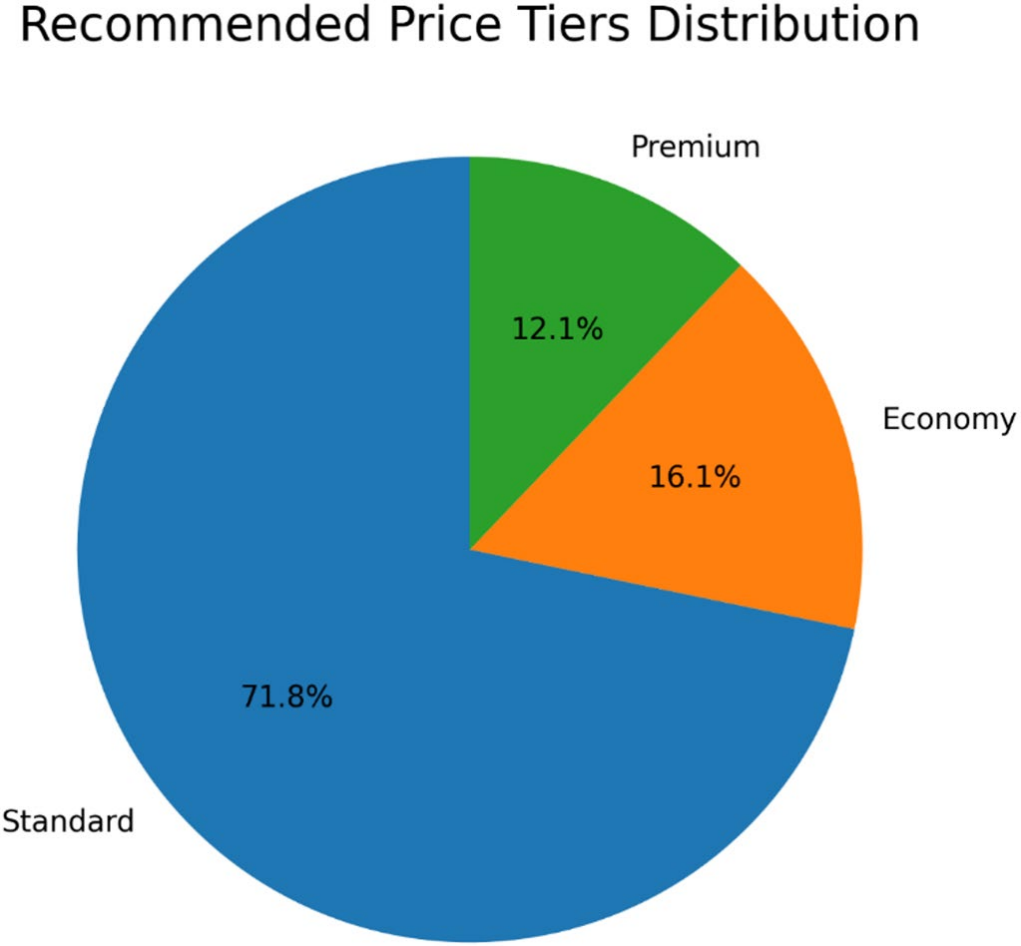
Distribution of recommended price tiers generated by the AI-driven pricing engine. The predominance of the Standard Tier reflects its alignment with the value proposition for the majority of the customer base, while the Economy and Premium tiers enable targeted segmentation.

The output of the dynamic pricing logic reveals a strategic segmentation of the customer base, as visualized in Fig. 13. The majority of customers (71.8%) were recommended the ‘Standard Tier,’ indicating that this option represents the optimal balance of value and features for the core customer segment. The smaller but distinct allocations to ‘Economy’ (16.1%) and ‘Premium’ (12.1%) tiers demonstrate the model’s capacity to identify price-sensitive customers and high-value targets, respectively. This data-driven stratification provides a empirical foundation for implementing differentiated pricing strategies that can maximize revenue and cater to diverse customer expectations within the CRM system.

**Fig. 14:**
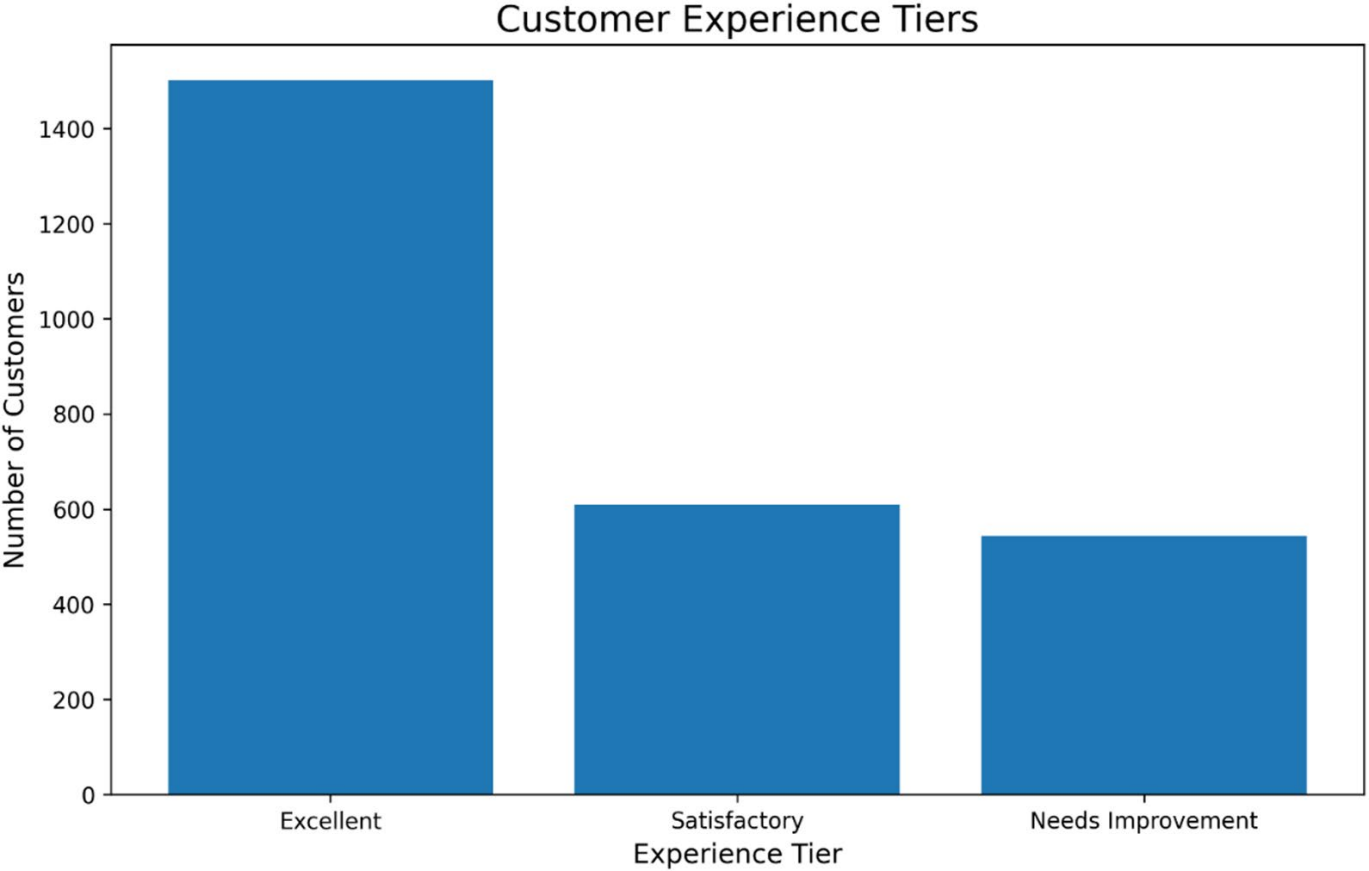
Customer distribution based on calculated experience score. While the majority report an excellent experience, the significant proportion in lower tiers identifies a key cohort for proactive service recovery and experience enhancement initiatives.

The distribution of customers across experience tiers, calculated from service interaction and usage data, provides a quantitative baseline for service quality assessment (Fig. 14). While a majority of customers fall into the ‘Excellent’ tier, a substantial cohort (approximately 1,100 customers) is classified as having ‘Satisfactory’ or sub-optimal experiences. This segmentation aligns with the predictive model’s finding that ‘customer service calls’ is a primary churn driver. The existence of this significant at-risk segment underscores the critical need for proactive intervention strategies targeted at improving the customer experience before dissatisfaction leads to attrition, thereby operationalizing the AI insights within a closed-loop CRM process.

**Fig. 15:**
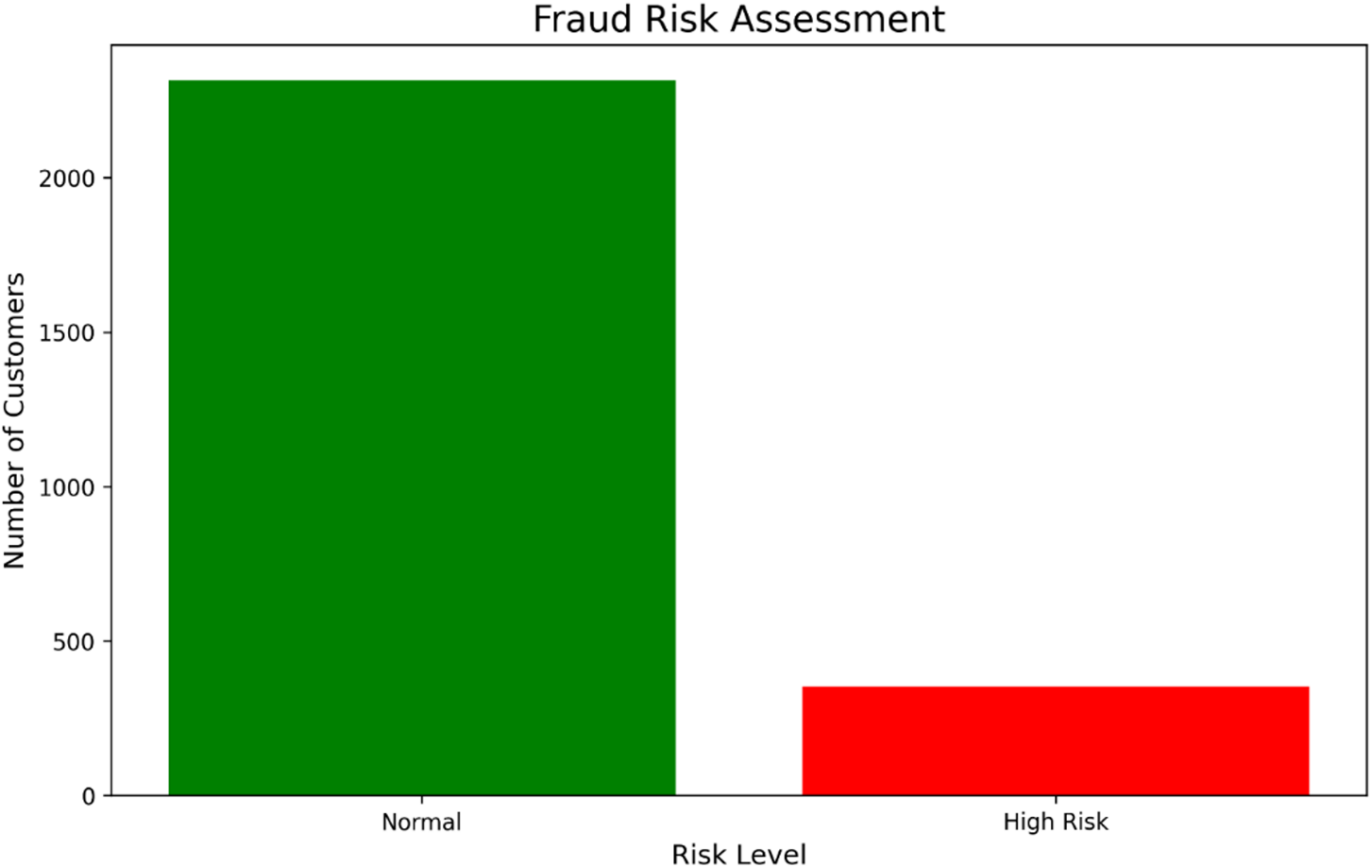
Customer classification based on fraud risk score derived from usage pattern anomalies. The distribution validates the model’s utility in identifying a focused subset of accounts for targeted investigation, enabling efficient resource allocation for fraud prevention.

The application of anomaly detection to customer usage patterns facilitates proactive risk management alongside churn prevention. As illustrated in Fig. 15, the model classified the majority of customers as ‘Normal Risk,’ affirming the integrity of the core customer base. However, it also identified a distinct, smaller cohort flagged as ‘High Risk.’ This segmentation enables a strategic, resource-efficient approach to security, allowing for intensified monitoring and verification procedures to be focused on a high-probability subset. This demonstrates how an integrated AI-CRM system can concurrently manage multiple business objectives—in this case, mitigating revenue loss from fraudulent activities without compromising the experience for the vast majority of legitimate customers.

**Fig. 16:**
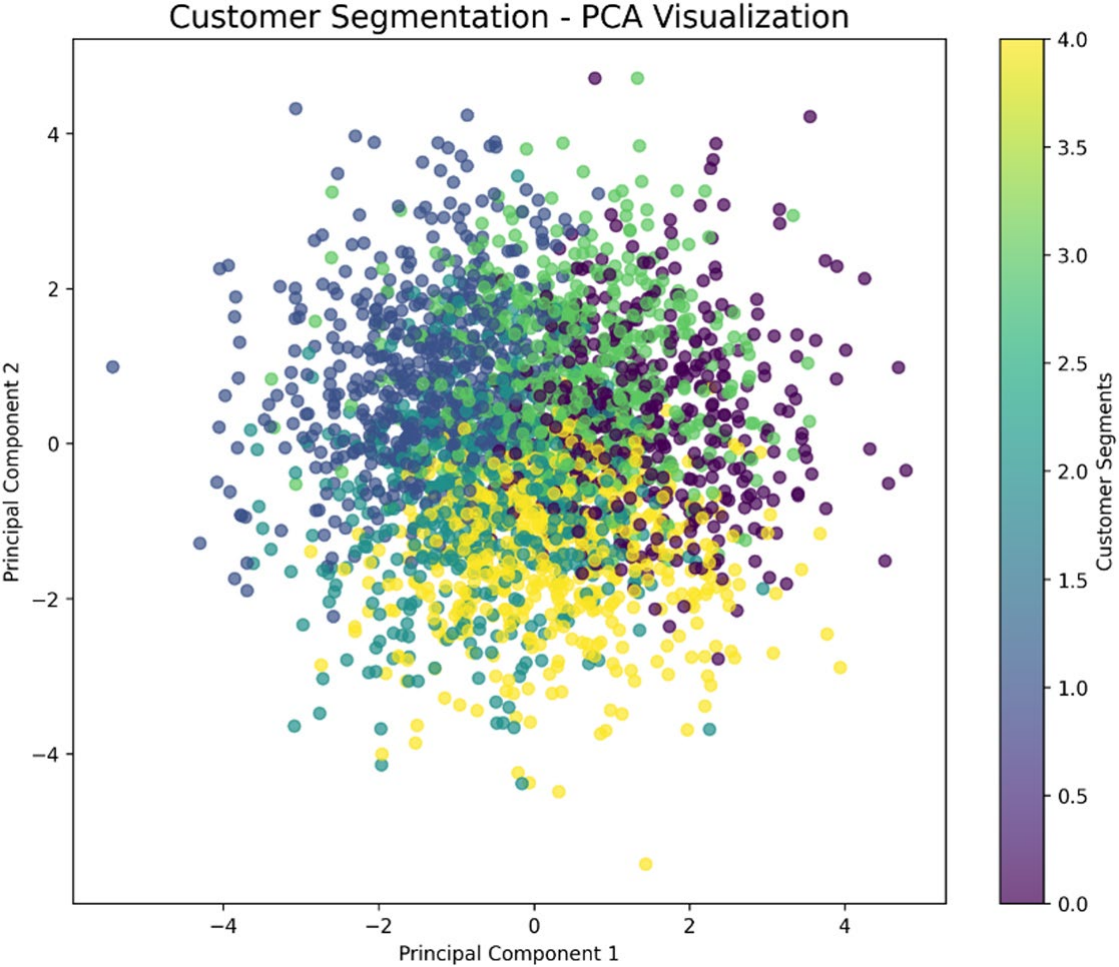
PCA visualization of customer segments derived from K-means clustering. The distinct clusters in the reduced principal component space represent unique customer cohorts based on service usage and behavioral patterns, enabling targeted relationship management strategies.

To complement the predictive model, customer segmentation was performed using K-means clustering on the feature set. The resulting segments, visualized via a two-dimensional Principal Component Analysis (PCA) projection in Fig. 16, reveal distinct, well-separated customer cohorts. The clear clustering in the reduced space validates that the underlying behavioral and usage data naturally separates customers into meaningful groups. These segments, ranging from potentially budget-conscious users to high-engagement premium customers, provide a strategic framework for moving beyond one-size-fits-all marketing to highly targeted, segment-specific CRM interventions, thereby personalizing the entire customer lifecycle.

**Fig. 17:**
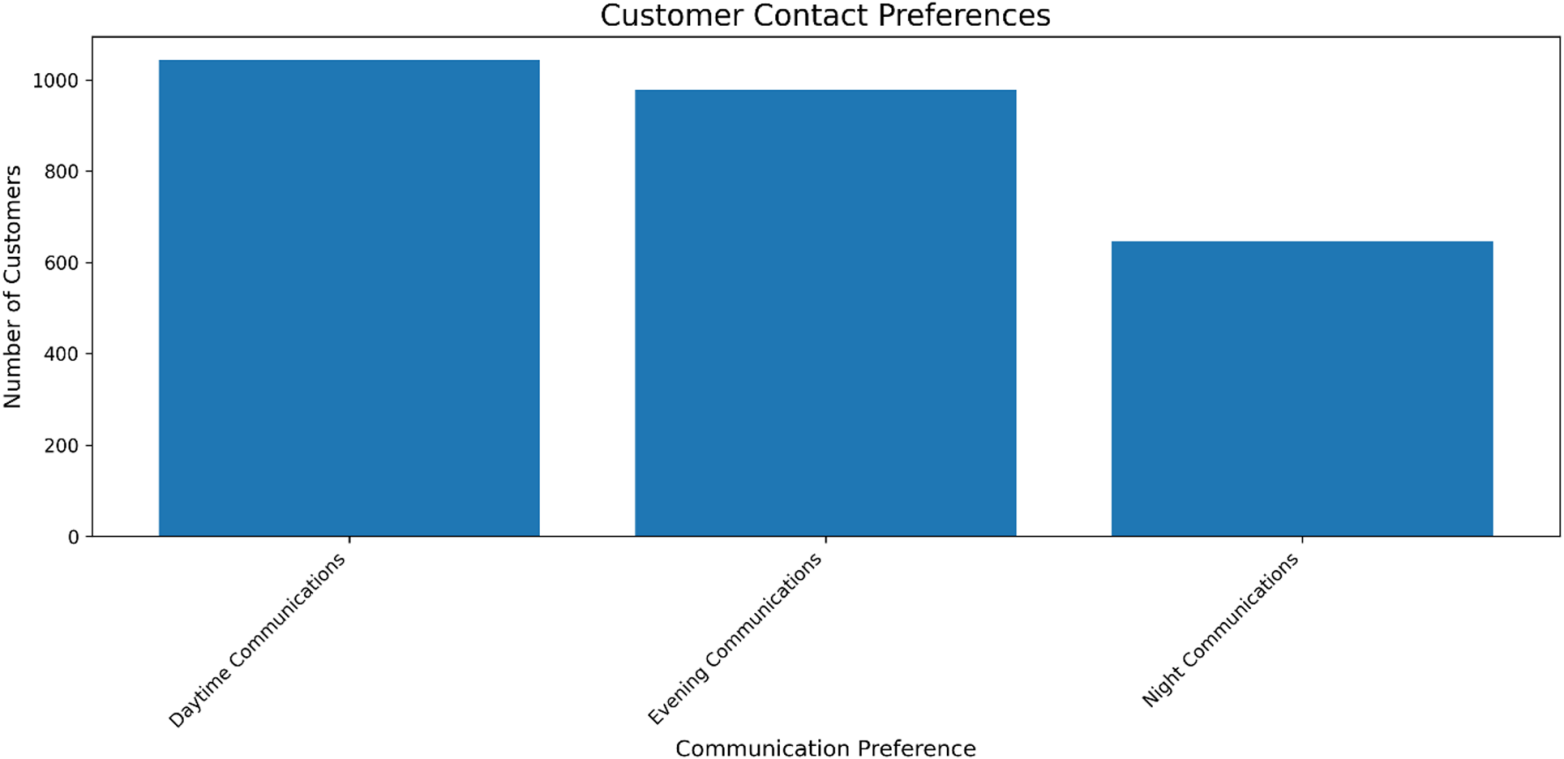
Distribution of inferred customer contact preferences based on behavioral data. The results inform a data-driven communication strategy, enabling the prioritization of daytime and evening outreach while maintaining reach to a significant night-preferring segment.

Analysis of inferred customer contact preferences, derived from engagement patterns, reveals a strategic opportunity for optimizing communication workflows (Fig. 17). While a majority of customers are most receptive during daytime and evening hours, a substantial segment shows a preference for night communications. This distribution provides an empirical basis for a multi-channel communication strategy that respects customer temporal preferences. Integrating these insights allows for the automation of contact scheduling within the CRM, ensuring that retention offers and service messages are delivered at times of highest likely engagement, thereby increasing campaign efficacy and improving the overall customer experience.

**Fig. 18:**
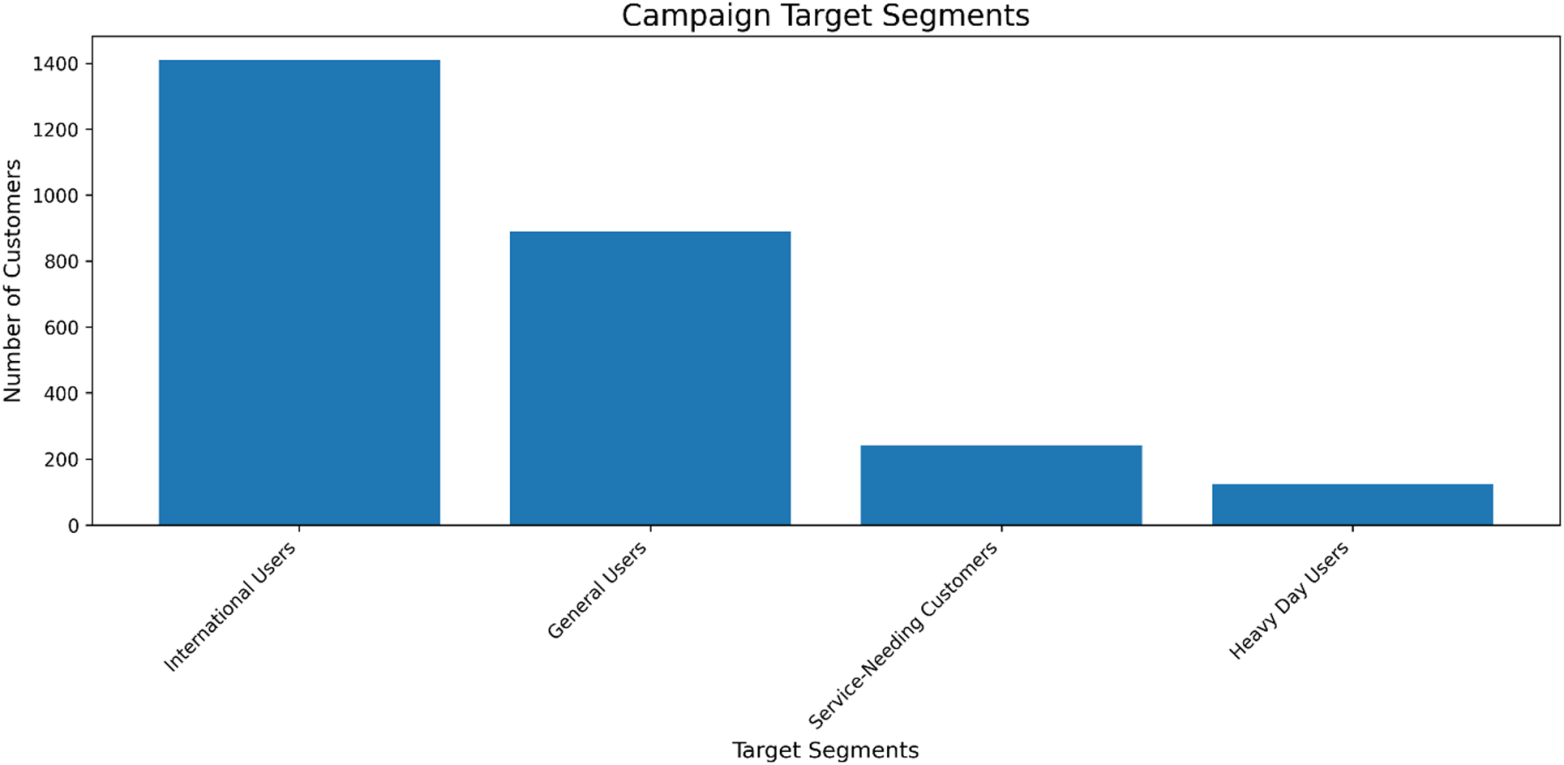
Size distribution of customer segments identified for targeted campaign management. The segments are defined by key behavioral attributes, enabling prioritized and tailored retention strategies based on segment size and strategic importance.

The segmentation of the customer base for targeted campaigns, as detailed in Fig. 18, provides an empirical basis for strategic resource allocation. The segmentation analysis identifies ‘International Users’ as the largest cohort, aligning with the feature importance of the ‘International Plan,’ followed by a substantial ‘General User’ segment. The smaller, but critically important, ‘Service Needing’ and ‘Heavy Day User’ segments correspond directly to the primary churn predictors identified by the model—customer service calls and daytime usage. This structured segmentation enables a precision marketing approach, where retention resources and communication strategies can be optimally tailored to the specific characteristics and risk profiles of each group.

While the model demonstrates high overall accuracy (95.13%) and excellent precision for the churn class (0.98), the recall of 0.68 indicates that 32% of actual churners were not identified. This reflects a classic trade-off in imbalanced classification. From a business perspective, this model is calibrated for high precision, ensuring that customers flagged as ‘high-risk’ are very likely to churn, thereby optimizing the cost-efficiency of retention campaigns. However, this comes at the expense of missing some true churners, a limitation that should be considered based on the specific cost-benefit analysis of retention actions.

The high accuracy (95.13%) can be attributed to the dataset’s clear feature separability and the effectiveness of the Random Forest algorithm in capturing the underlying patterns without overfitting, as confirmed by the strong performance on the held-out test set and the 10-fold cross-validation. Furthermore, the AUC of 0.89 provides a more robust evaluation metric, confirming the model’s strong discriminatory power.

Business Implications.

- Focus campaign efforts on International and General Users to maximize reach and impact.
- Consider tailored messaging or offers for Service Needing Customers to improve satisfaction.
- Explore the behavior of Heavy Day Users to understand their needs and potential value.
- The empirical results strongly support the research hypothesis. The high accuracy and AUC of the Random Forest model confirm that AI can reliably identify customers at risk of churning. The feature importance analysis provides actionable intelligence, revealing that efforts should be concentrated on customers with high daytime usage and those who frequently contact customer service.
- The strategic implications for telecom CRM are profound. We propose a multi-faceted approach:
- **(1) Proactive Retention Campaigns**: Deploy personalized retention campaigns targeting high-risk segments identified by the model. Offers could include tailored discounts, loyalty bonus minutes, or service upgrades based on individual usage patterns.
- **(2) Enhanced Customer Service Intervention**: Implement an alert system that flags customers who have made three or more service calls for proactive, high-touch support from a dedicated retention team, aiming to resolve underlying issues before they lead to churn.
- **(3) Service Quality and Plan Optimization**: Utilize insights from the model to address root causes of churn, such as network quality in areas with high daytime usage or redesigning international calling plans that are associated with attrition.
- **(4) Operationalization and Integration**: For maximum impact, the predictive model must be integrated directly into the company’s CRM and operational systems, enabling real-time scoring and triggering automated or semi-automated retention workflows.

Limitations and future research: This study has several limitations. First, the model was trained on a single, publicly available dataset, which may limit its generalizability to other telecom operators with different customer demographics and service structures. External validation on proprietary datasets is needed. Second, while we addressed class imbalance with SMOTE, the recall for the churn class could be further improved. Future work will explore advanced techniques like ensemble sampling and cost-sensitive learning. Third, the feature set was limited to structured data; incorporating unstructured data from customer calls and chats could enhance predictive power. Future research will also focus on real-time model deployment and A/B testing to quantitatively measure the causal impact of AI-driven interventions on churn reduction and customer lifetime value.

## Conclusion

Customer churn remains an ongoing challenge for telecom companies, and AI technologies offer a promising solution to address this. Through predictive analytics, AI can empower telecom companies to identify customers at risk of churn with accuracy, allowing for proactive and personalized interventions. This research aims to contribute to a deeper understanding of the role of AI in enhancing customer relationship management and reducing customer churn, providing valuable insights for practitioners and researchers in this field. This research conclusively demonstrates the efficacy of Artificial Intelligence, specifically the Random Forest algorithm, in predicting customer churn within the telecommunications sector. By achieving a high level of predictive accuracy and identifying key churn drivers, the study transformative impact for transforming CRM from a reactive to a proactive function. The integration of AI-powered predictive analytics allows companies to allocate resources efficiently, personalize customer interactions, and ultimately safeguard revenue. The novelty of this study lies not in the algorithm itself, but in its end-to-end framework that links explainable AI insights directly to actionable CRM strategies. By demonstrating how feature importance analysis can guide targeted interventions—such as focusing on customers with high daytime charges and multiple service calls—we provide a validated blueprint for telecom companies to move from predictive analytics to proactive customer retention, thereby enhancing the return on investment in AI technologies.

We recommend that telecom companies:

1. **Invest in AI Infrastructure**: Prioritize the development and integration of scalable AI and data analytics platforms within their existing CRM ecosystems.
2. **Adopt a Test-and-Learn Approach**: Continuously validate and refine predictive models with new data, and experiment with other algorithms like Gradient Boosting or explainable AI (XAI) models to further enhance performance and interpretability.
3. **Foster a Data-Centric Culture**: Encourage organizational practices where strategic decisions, particularly in marketing and customer service, are informed by predictive insights.
4. **Address Ethical Considerations**: Implement transparent data governance policies and ensure that automated decision-making processes are fair and unbiased, maintaining customer trust.

Future research could explore the use of deep learning models on larger datasets, incorporate unstructured data from customer interactions and social media, and longitudinally study the impact of these AI-driven interventions on long-term customer loyalty and lifetime value.

## Data Availability

Data will be available on request by contacting the corresponding author.
